# Prevention of UVB Induced Metabolic Changes in Epidermal Cells by Lipid Extract from Microalgae *Nannochloropsis oceanica*

**DOI:** 10.3390/ijms241411302

**Published:** 2023-07-11

**Authors:** Anna Stasiewicz, Tiago Conde, Agnieszka Gęgotek, Maria Rosário Domingues, Pedro Domingues, Elżbieta Skrzydlewska

**Affiliations:** 1 Department of Analytical Chemistry, Medical University of Bialystok, Kilinskiego 1, 15-069 Bialystok, Poland; anna.stasiewicz@umb.edu.pl (A.S.); elzbieta.skrzydlewska@umb.edu.pl (E.S.); 2 Mass Spectrometry Centre, LAQV-REQUIMTE, Department of Chemistry, University of Aveiro, Santiago University Campus, 3810-193 Aveiro, Portugal; tiagoalexandreconde@ua.pt (T.C.); mrd@ua.pt (M.R.D.); p.domingues@ua.pt (P.D.); 3 CESAM—Centre for Environmental and Marine Studies, Department of Chemistry, University of Aveiro, Santiago University Campus, 3810-193 Aveiro, Portugal

**Keywords:** UV radiation, microalgae, lipid extract, antioxidant enzymes, keratinocytes, inflammation

## Abstract

The exposure of skin cells to UV radiation leads to redox imbalances and inflammation. The present study investigates a lipid extract obtained from the microalga *Nannochloropsis oceanica* as a potential protector against UVB-induced disturbances in human keratinocytes. The findings of this study show that the *Nannochloropsis oceanica* extract significantly inhibits UVB-induced cell death while concurrently decreasing the activity of pro-oxidative enzymes (xanthine and NADPH oxidase) and reducing the levels of ROS. Furthermore, the extract augments the activity of antioxidant enzymes (superoxide dismutases and catalase), as well as glutathione/thioredoxin-dependent systems in UVB-irradiated cells. The expression of Nrf2 factor activators (p62, KAP1, p38) was significantly elevated, while no impact was observed on Nrf2 inhibitors (Keap1, Bach1). The antioxidant activity of the extract was accompanied by the silencing of overexpressed membrane transporters caused by UVB radiation. Furthermore, the *Nannochloropsis oceanica* extract exhibited anti-inflammatory effects in UVB-irradiated keratinocytes by decreasing the levels of TNFα, 8-iso prostaglandin F2, and 4-HNE-protein adducts. In conclusion, the lipid components of *Nannochloropsis oceanica* extract effectively prevent the pro-oxidative and pro-inflammatory effects of UVB radiation in keratinocytes, thereby stabilizing the natural metabolism of skin cells.

## 1. Introduction

The skin is the largest organ of the human body, responsible for protecting it from environmental aggressions, as well as contributing to the homeostasis of the organism [1]. The epidermis is a key player in these functions, serving as the first line of defence against microbial pathogens, toxic substances and physical factors such as ultraviolet (UV) radiation [2]. Every day, human epidermal cells are exposed to the UV radiation contained in sunlight. This is mainly highly penetrating UVA (315–400 nm) that reaches even deep parts of the skin, and high-energy UVB (280–320 nm) radiation, which is largely retained in the epidermal layers. Both of these factors affect the metabolism of the cells through which they penetrate; however, in the case of epidermal cells, UVB radiation is the main cause of irritation and erythema formation [3]. It is known that UVB can increase the production of reactive oxygen species (ROS), leading to oxidative stress and pro-inflammatory responses [4]. Simultaneously, endogenous antioxidant mechanisms, including enzymatic and non-enzymatic antioxidants, are activated. The expression of antioxidant proteins is regulated by redox-sensitive transcription factors, such as nuclear factor erythroid 2-related factor 2 (Nrf2), which regulates the expression of cytoprotective proteins, and nuclear factor kappa B (NFκB), which regulates inflammation [5,6]. However, under long-term oxidative stress, endogenous antioxidant systems can be overwhelmed [7]. The chronic exposure of epidermal cells to UVB radiation can result in hyperkeratosis, modifications in pigmentation, and structural changes in the skin, which not only trigger premature ageing [8,9], but also facilitate the onset of skin disorders such as eczema, atopic dermatitis, psoriasis, and vitiligo [10,11,12].

Due to the harmful effects of oxidative stress and UV-induced damage on skin cells, there is a growing interest in natural and exogenous compounds that possess antioxidant and anti-inflammatory properties. These natural compounds offer a promising alternative to synthetic compounds, which may be associated with undesirable side effects [13,14]. Algae, including macroalgae and microalgae, are a rich source of bioactive compounds, such as phospholipids, polyunsaturated fatty acids (PUFAs), polysaccharides and vitamins, and are being actively studied as potential modulators of the metabolism of skin cells [15,16]. Additionally, algae biomass and extracts, especially lipid extracts, are being evaluated for their therapeutic potential in various skin conditions [17,18], including thalassotherapy [14,19]. Additionally, algae oils enriched with omega-6 and omega-3 essential fatty acids, such as docosahexaenoic acid (DHA), are used in skin protection formulations [20].

So far, studies have demonstrated that lipid extracts obtained from microalgae such as *Chlorella vulgaris*, *Chlorococcum amblystomatis* and *Nannochloropsis oceanica* possess anti-inflammatory, antioxidant, antibacterial and antiproliferative properties [16,21]. Additionally, in vitro studies on macrophages (Raw264.7 and THP-1) have demonstrated that lipid extracts obtained from *Nitzschia palea*, *Chlorella vulgaris*, *Tetraselmis suecica*, *Micratinium* sp., *Aurantiochytrium mangrovei*, *Phaeodactylum tricornutum*, *Chloromona reticulata* and *Spirulina maxima* suppress the production of pro-inflammatory cytokines such as tumour necrosis factor alfa (TNFα), interleukins 1β and 6 (IL-1β and IL-6), which weaken the pro-inflammatory response and thus reduce inflammation [22,23,24]. One of the very promising microalgae species in human health protection is *Nannochloropsis oceanica*. *Nannochloropsis* species are green microalgae that are related to diatoms and brown algae. All of them are marine, except *N. limnetica*, which lives in freshwater. *Nannochloropsis oceanica*, which is a single-celled microalgae, was described for the first time in 2002 [25], and it is found in the Pacific Ocean, off the coast of Japan. It has long been used to produce nutraceuticals and feed supplements [26], according to the fact that the components of its cells may have therapeutic significance, especially in the context of supplementing the body with long-chain omega-3 polyunsaturated fatty acids or vitamin D3 [27,28,29]. In addition, *Nannochloropsis oceanica* is an effective functional food used against metabolic syndrome [30]. On the other hand, algal lipids have been shown to be modulators of skin diseases, and their pigment protects skin fibroblasts exposed to UVB radiation [31,32]. However, there is currently no clear understanding of the influence of microalgae lipid extracts on metabolic changes resulting from the action of UV rays on epidermal cells. The ability of lipid extracts from microalgae to modulate the effects of oxidative stress and inflammatory processes induced by UV radiation has not been studied to date. Understanding these regulatory mechanisms may be important for developing products for the daily protection of the skin against solar radiation, and in the treatment of the associated skin diseases mentioned above [33].

Therefore, the objective of this study is to analyze the impact of lipid extracts from the microalga *Nannochloropsis oceanica* on antioxidant and anti-inflammatory responses, in particular on the regulation of the activity of the transcription factors Nrf2 and NFκB in human keratinocytes exposed to UVB radiation.

## 2. Results

### 2.1. Viability of Keratinocytes

**Figure 1:**
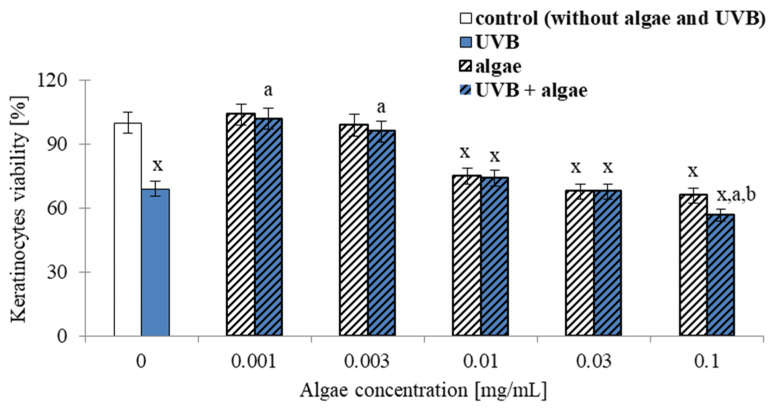
The impact of *Nannochloropsis oceanica* microalga lipid extracts on the viability of keratinocytes. The cells were subjected to varying concentrations of the extract (0–0.1 mg/mL) for 24 h, with UVB exposure (60 mJ/cm^2^, UVB and UVB + algae) and without UVB exposure (control and algae). The control group was not subjected to UVB or algae extract. The mean values ± standard deviation (n = 5 for each group) are presented. The statistically significant differences are denoted as follows: “x” indicates a comparison to the control group (control vs. all groups); “a” indicates a comparison to the group of keratinocytes that were exposed only to UVB (UVB group); “b” indicates a comparison to the group of keratinocytes that were exposed to the same concentration of microalgae extract (algae vs. algae + UVB). Groups with the same letter indicate significant differences between them (*p* < 0.05).

The results obtained from the MTT assay demonstrate that the viability of both unexposed and irradiated keratinocytes was affected in a concentration-dependent manner by the microalga *Nannochloropsis oceanica* lipid extract used in the experiment. Specifically, extract concentrations of 0.001 and 0.003 µg/mL had no significant impact on the cell viability of unirradiated keratinocytes, whereas higher concentrations of the seaweed extract (≥0.01 mg/mL) reduced keratinocyte viability. At a concentration of 0.1 mg/mL, cell viability was decreased to approximately 65% (Figure 1).

Keratinocytes subjected to UVB radiation alone exhibited a reduction in cell viability of approximately 30%. In contrast, exposure to both UVB radiation and low concentrations of microalgae lipid extract (≤0.003 mg/mL) resulted in a significant increase in cell viability, indicating that the lipids can protect the cells from the harmful effects of UVB radiation and promote cell survival up to nearly 100%. However, when higher concentrations of the seaweed extract (≥0.01 mg/mL) were used, the survival rate of irradiated keratinocytes decreased, although it was still higher than that observed with UVB exposure alone. At a concentration of 0.1 mg/mL of the algal extract in cells exposed to UVB, no significant difference in cell viability was observed compared to UVB exposure alone.

### 2.2. Keratinocyte Membrane Transporters

**Figure 2:**
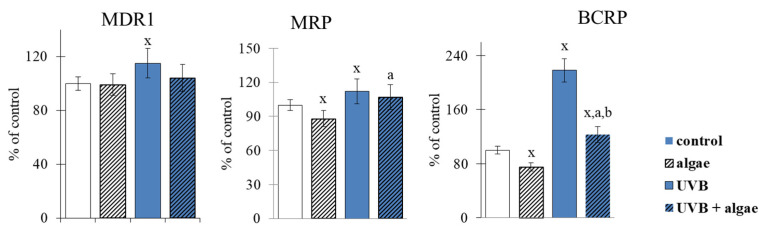
The effect of lipid extract from microalga *Nannochloropsis oceanica* (0.003 mg/mL) on the activity of ABC transporters (MDR1, MRP, BCRP) in keratinocytes. Four experimental groups were analyzed: control (not treated with the lipid extract or UVB), algae (exposed to the lipid extract for 24 h), UVB (exposed to UVB radiation (60 mJ/cm^2^)) and UVB + algae (exposed to UVB radiation and then treated with the lipid extract for 24 h). The data are expressed as a percentage relative to the control group and presented as mean values ± SD (n = 5 for each group). The statistically significant differences are denoted as follows: “x” indicates a comparison to the control group (control vs. all groups); “a” indicates a comparison to the algae group (all groups vs. the algae group); “b” indicates a comparison to the UVB group (all groups vs. the UVB). Groups with the same letter indicate significant differences between them (*p* < 0.05).

The impact of *Nannochloropsis oceanica* microalga lipid extract on keratinocyte membrane transporters was also investigated. It was determined that the lipid extract did not affect the activity of the MDR1 keratinocyte membrane transporter. However, incubating cells with the lipid extract resulted in a significant reduction in the activity of two other ATP-binding cassette (ABC) transporters, namely, MRP and BCRP. In contrast, UVB radiation increased the activity of all analyzed transporters, with the BCRP transporter showing the highest increase (approximately a 2.5-fold increase) (Figure 2). When keratinocytes were exposed to UVB radiation and the algae lipid extract, no significant differences were observed in the activity of MDR1 and MRP transporters, but a notable decrease (by approximately 50%) in the activity of the BCRP transporter was observed.

### 2.3. Redox Balance of Keratinocytes

**Figure 3:**
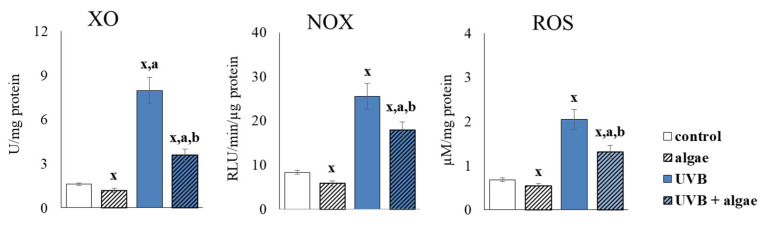
The effect of lipid extract from microalga *Nannochloropsis oceanica* (0.003 mg/mL) on the activity of xanthine oxidase (XO), NADPH oxidase (NOX) and reactive oxygen species (ROS) in keratinocytes. Four experimental groups were analyzed: control (not treated with the lipid extract or UVB), algae (exposed to the lipid extract for 24 h), UVB (exposed to UVB radiation (60 mJ/cm^2^)) and UVB + algae (exposed to UVB radiation and then treated with the lipid extract for 24 h). The data are expressed as a percentage relative to the control group and presented as mean values ± SD (n = 5 for each group). The statistically significant differences are denoted as follows: “x” indicates a comparison to the control group (control vs. all groups); “a” indicates a comparison to the algae group (all groups vs. the algae group); “b” indicates a comparison to the UVB group (all groups vs. the UVB). Groups with the same letter indicate significant differences between them (*p* < 0.05).

The supplementation of keratinocytes with a lipid extract from *Nannochloropsis oceanica* (0.003 mg/mL) for 24 h was found to reduce the activity of the cytosolic enzymes responsible for superoxide anion generation, namely, xanthine oxidase (XO) and NADPH oxidase (NOX), resulting in an approximately 20% decrease in total ROS levels (Figure 3). UVB radiation exposure resulted in approximately 5- and 3-fold increases in XO and NOX activity, respectively, and more than a 2-fold increase in ROS levels. The supplementation of UVB-irradiated cells with the microalgae lipid extract resulted in the significant prevention of an increase in the activity of pro-oxidative enzymes (by approximately 60% for XO and approximately 40% for NOX) and the level of ROS (by approximately 40%).

**Figure 4:**
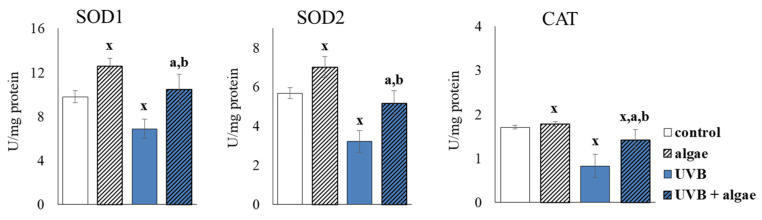
The effect of lipid extract from microalga *Nannochloropsis oceanica* (0.003 mg/mL) on the activity of cytosolic and mitochondrial superoxide dismutase (SOD1 and SOD2) and catalase (CAT) in keratinocytes. Four experimental groups were analyzed: control (not treated with the lipid extract or UVB), algae (exposed to the lipid extract for 24 h), UVB (exposed to UVB radiation (60 mJ/cm^2^)) and UVB + algae (exposed to UVB radiation and then treated with the lipid extract for 24 h). The data are expressed as a percentage relative to the control group and presented as mean values ± SD (n = 5 for each group). The statistically significant differences are denoted as follows: “x” indicates a comparison to the control group (control vs. all groups); “a” indicates a comparison to the algae group (all groups vs. the algae group); “b” indicates a comparison to the UVB group (all groups vs. the UVB). Groups with the same letter indicate significant differences between them (*p* < 0.05).

The lipid extract obtained from *Nannochloropsis oceanica*, when added to the culture medium of keratinocytes, resulted in an increase in the activity of fundamental cellular enzymes that are responsible for the metabolism of superoxide anions. Specifically, the mitochondrial and cytosolic superoxide dismutase (Mn-SOD and Cu, Zn-SOD) were activated by 24% and 28%, respectively. It was observed that in cells exposed to UVB irradiation, there was a reduction in the activity of both isoforms of SOD and CAT. However, this trend was significantly prevented when the algae extract was administered to cells treated with UVB, as shown in Figure 4.

**Figure 5:**
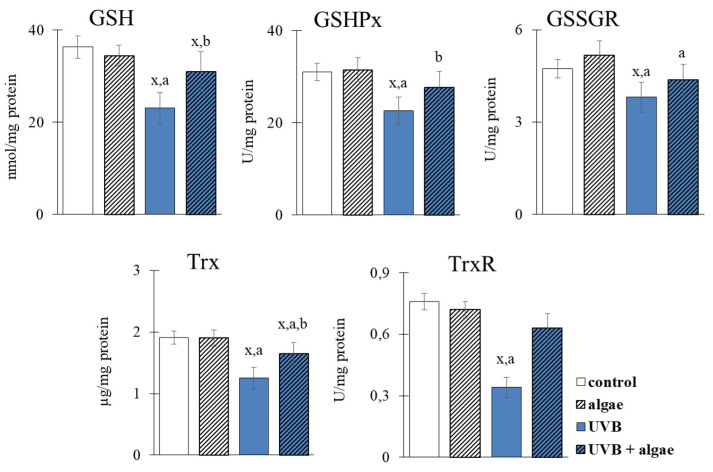
The effect of lipid extract from microalga *Nannochloropsis oceanica* (0.003 mg/mL) on the activity of the GSH-dependent antioxidant system (GSH, GSHPx, GSSGR) and Trx-dependent antioxidant system (Trx, TrxR) in keratinocytes. Four experimental groups were analyzed: control (not treated with the lipid extract or UVB), algae (exposed to the lipid extract for 24 h), UVB (exposed to UVB radiation (60 mJ/cm^2^)) and UVB + algae (exposed to UVB radiation and then treated with the lipid extract for 24 h). The data are expressed as a percentage relative to the control group and presented as mean values ± SD (n = 5 for each group). The statistically significant differences are denoted as follows: “x” indicates a comparison to the control group (control vs. all groups); “a” indicates a comparison to the algae group (all groups vs. the algae group); “b” indicates a comparison to the UVB group (all groups vs. the UVB). Groups with the same letter indicate significant differences between them (*p* < 0.05).

The introduction of the microalgae extract into the keratinocyte medium did not demonstrate any significant effects on the levels of glutathione (GSH) and thioredoxin (Trx), as well as enzymes such as glutathione peroxidase (GSHPx), glutathione-disulfide reductase (GSSG-R), and thioredoxin reductase (TrxR) (Figure 5). However, UVB radiation exposure to the keratinocytes significantly decreased the efficacy of both antioxidant systems responsible for protecting the cells from the effects of oxidative stress, i.e., the GSH-dependent and Trx-dependent systems. In contrast, the utilization of the microalgae lipid extract noticeably counteracted the decrease induced by UVB in the level/activity of the components of both systems, ranging from 15% for GSSG-R to 85% for TrxR, indicating the protective impact of the microalgae lipid extract on oxidative stress effects in keratinocytes.

**Figure 6:**
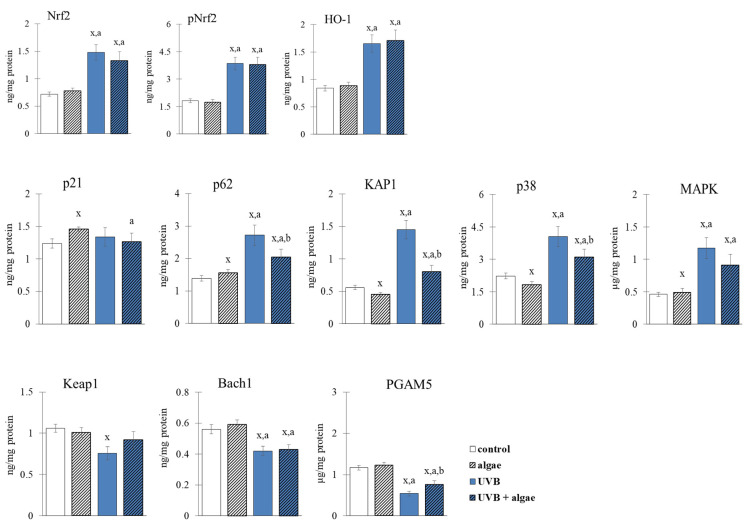
The effect of lipid extract from microalga *Nannochloropsis oceanica* (0.003 mg/mL) on the expression of transcription factor Nrf2 and its main product (HO-1), its inhibitors (Keap1, Bach1, PGAM5) and its activators (p21, p62, KAP1, p38. MAPK) in keratinocytes. Four experimental groups were analyzed: control (not treated with the lipid extract or UVB), algae (exposed to the lipid extract for 24 h), UVB (exposed to UVB radiation (60 mJ/cm^2^)) and UVB + algae (exposed to UVB radiation and then treated with the lipid extract for 24 h). The data are expressed as a percentage relative to the control group and presented as mean values ± SD (n = 5 for each group). The statistically significant differences are denoted as follows: “x” indicates a comparison to the control group (control vs. all groups); “a” indicates a comparison to the algae group (all groups vs. the algae group); “b” indicates a comparison to the UVB group (all groups vs. the UVB). Groups with the same letter indicate significant differences between them (*p* < 0.05).

**Figure 7:**
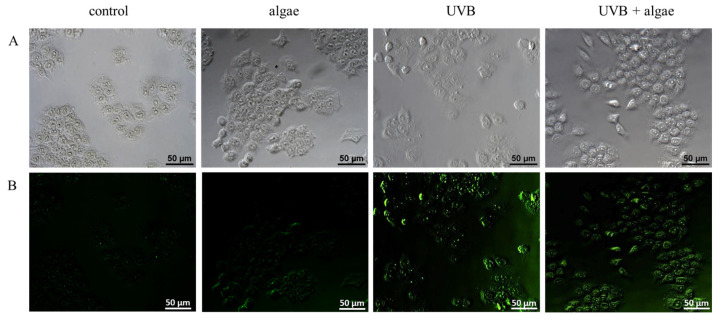
The impact of a 24 h incubation of keratinocytes with a lipid extract derived from *Nannochloropsis oceanica* microalgae (0.003 mg/mL) on the intracellular distribution of the Nrf2 transcription factor in cells exposed to UVB radiation (60 mJ/cm^2^). (**A**) Microscopic photos; (**B**) localization of Nrf2, which was stained green.

The alterations in the redox equilibrium of keratinocytes may be attributed to the potential impact of the *Nannochloropsis oceanica* microalgae extract on the activity of the Nrf2 transcription factor, which controls the synthesis of antioxidant proteins. In non-irradiated keratinocytes, the microalgae extract did not elicit any effect on the expression or localization of the Nrf2 factor, or its downstream effector molecule, heme oxygenase 1 (HO-1) (Figure 6 and Figure 7). Conversely, exposure to UVB radiation increased the expression and nuclear translocation of Nrf2 or its phosphorylated form (pNrf2), thereby increasing the levels of heme oxygenase. The observed alterations in Nrf2 transcription following UVB irradiation may result from the markedly reduced levels of all assessed Nrf2 inhibitors, such as KEAP1, Bach1, and PGAM5, and the significantly elevated levels of most activators, including p62, KAP1, p38, and MAPK (Figure 6).

Using the lipid extract from microalgae prevented the UVB-induced reduction in Keap1 levels, as well as a significant reduction in the activators p62, KAP1, and p38 (Figure 6). Consequently, the level of expression and transcriptional efficiency of Nrf2 assessed via the level of HO-1 did not change in comparison to keratinocytes exposed to UVB. However, the lipid extract did not significantly affect Nrf2 expression in irradiated keratinocytes, but it did alter its intracellular location, partially preventing its UVB-induced translocalization to the cell nucleus (as shown in Figure 7).

### 2.4. Anti-Inflammatory Effect of Lipid the Extract

**Figure 8:**
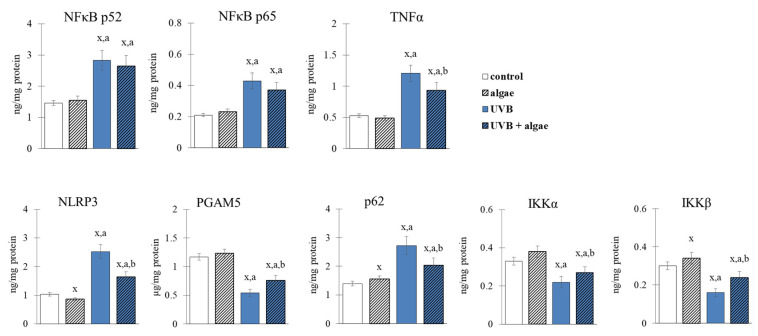
The effect of lipid extract from microalga *Nannochloropsis oceanica* (0.003 mg/mL) on the expression of NFκB (p52 and p65) and the main cytokine (TNFα), its inhibitors (IKKα, IKKβ) and its activators (NLRP3, PGAM5, p62) in keratinocytes. Four experimental groups were analyzed: control (not treated with the lipid extract or UVB), algae (exposed to the lipid extract for 24 h), UVB (exposed to UVB radiation (60 mJ/cm^2^)) and UVB + algae (exposed to UVB radiation and then treated with the lipid extract for 24 h). The data are expressed as a percentage relative to the control group and presented as mean values ± SD (n = 5 for each group). The statistically significant differences are denoted as follows: “x” indicates a comparison to the control group (control vs. all groups); “a” indicates a comparison to the algae group (all groups vs. the algae group); “b” indicates a comparison to the UVB group (all groups vs. the UVB). Groups with the same letter indicate significant differences between them (*p* < 0.05).

**Figure 9:**
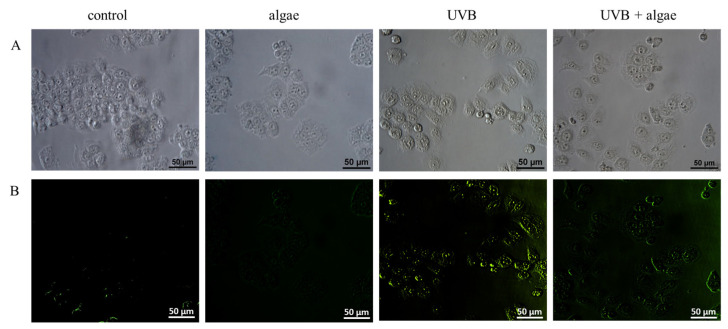
The impact of a 24 h incubation of keratinocytes with a lipid extract derived from *Nannochloropsis oceanica* microalgae (0.003 mg/mL) on the intracellular distribution of the transcription factor NFκB(p52) in cells exposed to UVB radiation (60 mJ/cm^2^). (**A**) Microscopic photos; (**B**) localization of transcription factor NFκB(p52), which was stained green.

The microalga extract induced changes in the NFκB transcription factor pathway (shown in Figure 8 and Figure 9) similar to those observed with Nrf2. The results demonstrate a decrease in the levels of NLRP3 and p62 activators, and one of its inhibitors (IKKβ), resulting in an absence of response in the expression of p52 and p65 NFκB subunits, and the transcriptional product TNFα (Figure 8). In keratinocytes exposed to UVB radiation, the levels of NFκB activators (NLRP3 and p62) and the IKKα and IKKβ inhibitors were significantly increased. Treatment with the lipid microalga extract significantly prevented the UVB-induced nuclear translocation of NFκB(p52), as well as the increase in the level of activators, while resulting in an increase in the level of inhibitors. As a consequence, the levels of NFκB subunits did not change significantly, but the level of cytokine TNFα was decreased.

### 2.5. Lipid Peroxidation in Keratinocytes

**Figure 10:**
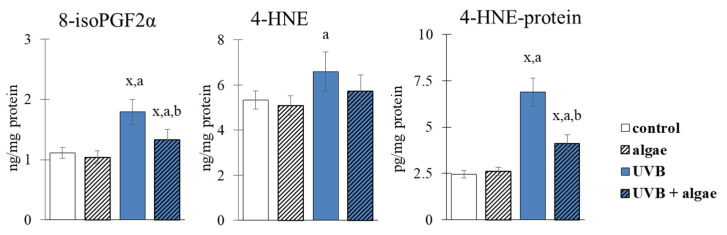
The effect of lipid extract from microalga *Nannochloropsis oceanica* (0.003 mg/mL) on the levels of lipid peroxidation products (8-isoPGF2α, 4-HNE) and HNE-protein adducts in keratinocytes. Four experimental groups were analyzed: control (not treated with the lipid extract or UVB), algae (exposed to the lipid extract for 24 h), UVB (exposed to UVB radiation (60 mJ/cm^2^)) and UVB + algae (exposed to UVB radiation and then treated with the lipid extract for 24 h). The data are expressed as a percentage relative to the control group and presented as mean values ± SD (n = 5 for each group). The statistically significant differences are denoted as follows: “x” indicates a comparison to the control group (control vs. all groups); “a” indicates a comparison to the algae group (all groups vs. the algae group); “b” indicates a comparison to the UVB group (all groups vs. the UVB). Groups with the same letter indicate significant differences between them (*p* < 0.05).

The evaluation of lipid peroxidation in keratinocytes revealed that the lipid extract derived from *Nannochloropsis oceanica* did not cause oxidative damage to PUFAs, as there were no observable changes in the levels of electrophilic aldehyde (4-HNE) and iso-prostaglandin (8-isoPGF2α) (Figure 10). However, exposure of the cells to UVB radiation resulted in an increase in the levels of both lipid peroxidation products. The addition of the lipid extract from microalgae to the culture medium after exposure to UVB radiation significantly decreased the level of 8-isoPGF2α. The lipid extract also decreased the levels of 4-HNE adducts with nucleophilic proteins induced by UVB radiation, by 40%.

## 3. Discussion

The human skin is frequently exposed to solar radiation, which includes UVB radiation, and this exposure can alter the metabolism of keratinocytes—the fundamental cells of the epidermis [34]. Therefore, there is a constant pursuit of natural substances and compounds to protect the skin against the adverse effects of solar radiation. Prior research suggests that UVB radiation can have a pro-oxidative effect on keratinocytes, leading to an increase in the activity of ROS-generating enzymes such as NOX and XO [35,36]. This pro-oxidative effect is also accompanied by a decrease in the antioxidant capacity of keratinocytes, as cellular antioxidant enzymes, such as cytosolic and mitochondrial superoxide dismutase, and the glutathione-dependent and thioredoxin-dependent systems, are reduced, thereby limiting their ability to counteract the increased generation of ROS and protect cellular components [37,38]. Consequently, oxidative stress is observed in keratinocytes exposed to UVB, resulting in the oxidative modification of small- and high-molecular components, including lipids and proteins [34]. Of note, phospholipids and free PUFAs are particularly susceptible to modification, with the generation of small-molecular weight α,β-unsaturated reactive aldehydes, such as 4-HNE [39], and the formation of cyclic derivatives of prostaglandins, including neuroprostanes and isoprostanes, through the oxidative cyclization of PUFAs [40].

The presence of oxidative stress induced by UVB radiation activates enzymes that metabolize polyunsaturated fatty acids (PUFAs) and affects the levels of other lipid mediators, such as endocannabinoids and eicosanoids [37]. This leads to the activation of receptors associated with G protein and the promotion of an inflammatory response by increasing the levels of pro-inflammatory interleukins, including TNFα, and disrupting the balance of redox stress. Consequently, the metabolism of keratinocytes is altered, resulting in reduced expression of the cytoprotective protein biosynthesis transcription factor Nrf2 and increased expression of the inflammatory process, promoting the transcription factor NFκB [41]. The results from this study and data from the literature suggest that UVB radiation modifies the metabolism of epidermal cells by activating oxidative and inflammatory processes, which could lead to accelerated skin ageing and the onset of disease processes [42].

To explore the potential of ameliorating the observed metabolic alterations, a lipid extract derived from the microalga *Nannochloropsis oceanica* was administered to keratinocytes. Initially, we evaluated the impacts of various concentrations of the extract on cell viability to ascertain the appropriate concentration for subsequent investigations. The outcomes reveal that the most favorable concentration was 0.03 mg/mL (Figure 1), since it reverted the viability to the same levels as the control keratinocytes.

The detailed lipid composition of the extract used has been published previously [43]. It is a product rich in glycolipids, especially mono- and diacylglyceryl 3-O-4′-(N,N,N-trimethyl) homoserine, sulfoquinovosyldiacylglycerol, mono- and digalactosyldiacylglycerol (MDGD and DGDG), but also in phospholipids and neutral lipids. The most significant components in the group of phospholipids are phosphatidylcholine, phosphatidylethanolamine, phosphatidylglycerol and phosphatidylinositol. This rich lipid composition prompted the search for its most effective activities in cell membranes. Moreover, high concentrations of sfingolipids and ceramides specialize this extract to the interaction with skin cells whose natural intercellular substance also contains these components [44,45]. Therefore, the effect of keratinocytes supplementation with microalgae extract was determined by assessing the activity of membrane transporters (ABC). The partial inhibition of MRP and BCRP was observed following the administration of the extract (Figure 2). Conversely, the UVB irradiation of keratinocytes, likely due to oxidative modifications of lipids and membrane proteins, increased the activity of ABC transporters, including BCRP, several-fold. These results are in agreement with a previous study showing that oxidative stress results in the upregulation of ABC transporters, such as MDR1, MRP1, MRP2, MRP4, and BCRP [46]. Our findings suggest that the activity of the BCRP transporter is significantly reduced by approximately 20% after supplementation with the microalga extract (Figure 2). The expression and transport functions of proteins are regulated by several factors, including the Nrf2 transcription factor, which is modulated by the p53, p38, and NFκB proteins [47]. Based on the available data, it is challenging to unequivocally determine which transporters are involved in transporting the components of the microalga extract through the cell membrane. Furthermore, considering that the activity of the BCRP transporter is enhanced after the irradiation with UVB and the supplementation with the microalga extract, this could improve the transport of these components across keratinocyte membranes. It is known that lipid extracts from algae contain numerous compounds with antioxidant properties, such as PUFAs and low-molecular weight lipophilic compounds [24,48,49,50,51,52,53], which may have a protective effect after entering cells.

The results of this study support the effectiveness of the protective properties of the lipid extract derived from *Nannochloropsis oceanica* when incorporated into keratinocyte cultures. This was evidenced by a decrease in levels of UVB-induced NOX and ROS, which can be attributed to the diminished activity of pro-oxidative enzymes, such as xanthine oxidase and NADPH oxidase (Figure 3). Other studies have also shown that extracts obtained from algae, such as *Carpomitra costata* and *Ettlia* sp., can reduce superoxide anion and hydroxyl radical levels in human keratinocytes and fibroblasts, thus underscoring the potential of algae-based components to mitigate ROS levels [54,55]. Previously, the literature has primarily attributed the antioxidant capacity of algae to phenolic compounds and lipids, particularly ω-3 PUFAs [56,57,58,59]. Furthermore, a sulfolipid fraction isolated from *Porphyridium cuentum* was shown to suppress superoxide anion radical production in leukocytes treated with phorbol myristate acetate [60]. MGDG and DGDG interacting with ROS can also directly reduce their levels [61]. In addition, astaxanthin, a carotenoid present in algae, has been increasingly indicated as a compound that reduces the activity of pro-oxidative enzymes such as xanthine oxidase and NADPH oxidase [62]. The observed reduction in ROS generation, after supplementation with the *Nannochloropsis oceanica* extract, suggests that there is an enhanced antioxidant efficacy in both control keratinocytes and those exposed to UVB radiation, through the increased activity of important antioxidant enzymes such as cytosolic and mitochondrial superoxide dismutase (Cu,Zn-SOD and Mn-SOD) (Figure 4). The augmented activity of MnSOD, in particular, decreases the probability of electron leakage from the mitochondrial chain, and therefore prevents the generation of ROS.

On the contrary, the use of microalgae shifts the redox balance towards reducing conditions, which leads to the increased effectiveness of interacting antioxidant systems. These systems include the GSH-dependent and Trx-dependent systems, which require reducing conditions to effectively participate in the protective effects of lipid PUFAs and the reduction in disulfide bridges of peptides and proteins [63]. However, we observed only a partial restoration of GSH levels to control cell levels after supplementation with the extract (Figure 5), possibly due to the formation of stable conjugates of GSH with 4-hydroxynonenal (4-HNE). 4-HNE is an active electrophilic aldehyde formed due to cell lipid peroxidation after the irradiation of UVB cells [39], and its level was not normalized even after the use of *Nannochloropsis oceanica* lipid extract. This mechanism is supported by the significantly increased level of 4-HNE-protein adducts after the treatment with UVB (Figure 10).

The interaction of GSH with glutathione peroxidase serves as a protective mechanism for membrane phospholipids in keratinocytes [64]. However, the literature data suggest that the reduced efficiency of the GSH-dependent system is counteracted by the Trx-dependent system, which exhibits a dominant antioxidant effect in the cytosol [65]. This study also observed the increased effectiveness of the Trx-dependent system after the use of microalgae extract (Figure 5). Algae lipid extracts, such as those from *N. oceanica*, exhibit significant antioxidant effects due to their high content of phospholipids rich in PUFAs, including ω-3 FAs [66]. Furthermore, the results of this study demonstrate a significant prevention of increased lipid peroxidation, as shown by decreased levels of the product of lipid peroxidation (4-HNE) and cyclization (8-isoPGF2α) after incubation with the lipid extract of cells treated with UVB (Figure 10). The components of lipid extracts from algae, including sterols, have been found to inhibit the expression of enzymes, including metalloproteinases, and proteins involved in the regulation of oxidative stress by modulating the MAPKs of UV-irradiated keratinocytes [67].

The cellular response to oxidative stress involves various transcription factors, notably Nrf2, which is responsible for the biosynthesis of cytoprotective proteins, and NFκB, which mediates the biosynthesis of pro-inflammatory proteins and partially regulates the Nrf2 response [68,69]. The supplementation with the *Nannochloropsis oceanica* extract did not significantly influence the augmented expression of Nrf2 after UVB treatment (Figure 6). However, microscopy has shown evidence that the microalgae lipid extract partially prevented the translocalization induced by UVB to the cell nucleus (Figure 7). Nevertheless, it is known that specific receptors for growth factors, i.e., keratinocyte growth factor (KGF), are expressed on the surfaces of keratinocytes and have the potential to activate Nrf2 without triggering its biosynthesis [70]. Additionally, carotenoids, such as lipophilic astaxanthin, are present in this microalga [71]. Astaxanthin has significant antioxidant activity on irradiated skin fibroblasts, upregulating the expression of classical antioxidant enzymes such as HO-1, superoxide dismutase 2 (SOD2), catalase (CAT), and glutathione peroxidase (GSHPx) through Nrf2 activation [72,73]. This sustains the elevated Nrf2 expression in UVB-exposed keratinocytes and microalgae. Moreover, the phosphatidylinositol contained in large amounts in the used lipid extract may directly induce a phosphatidylinositol 3-kinase dependent pathway of Nrf2 activation [74]. Also, in vivo experiments have demonstrated that astaxanthin amplifies the mRNA levels of heme oxygenase-1 (HO-1) and glutathione S-transferase (GST), and promotes the enhanced expression of these proteins at the post-transcriptional level [75]. Therefore, the elevated levels of Nrf2 and HO-1 (Figure 6), as well as the higher activities of other antioxidant enzymes, could be a biological consequence of this carotenoid present in the algal extract. Among the lipid components of microalgae, fatty acids, particularly omega-3 polyunsaturated fatty acids (ω-3 PUFAs), have also been attributed with antioxidant activity [66]. Furthermore, a previous study indicated that the ethanol extract of the macroalga *Carpomitra costata* promoted an increase in the level of antioxidants in keratinocytes exposed to UVB [54].

Under normal physiological conditions, the cytoplasmic Nrf2 protein is bound to Keap1, which facilitates its ubiquitination and subsequent proteasomal degradation [76]. The microalga extract seems to enhance the inhibitory effects of Keap1 by decreasing the levels of ROS and 4-HNE, which are known to increase after exposure to UVB radiation, and by possibly reducing the number of oxidized cysteine residues on this protein. This is crucial for the biological activity of Keap1 as an inhibitor [77]. However, the use of the microalgae extract did not affect the expression of the nuclear inhibitor Bach1 (Figure 6), which was reduced following exposure to UVA radiation. In contrast, the use of the microalgae lipid extracts partially prevented the downregulation of PGAM5 (Figure 6), another Nrf2 inhibitor [78], following exposure to UVA. 

Other studies have reported that the transcriptional activity of Nrf2 can be further improved by elevated levels of prostaglandin 15d-PGJ2, which can form adducts with Keap1 [77]. The anticipated elevation in the level of 15d-PGJ2 may also be related to oxidative stress in keratinocytes, which induces the activation of lipolytic enzymes (PLA2 and COXs). The effects of these enzymes on PUFAs present in the *Nannochloropsis oceanica* lipid extract, which are precursors of eicosanoids, including prostaglandin 15d-PGJ2 [79], should also be considered. Consequently, Nrf2 is separated from the Keap1 adduct and translocated to the nucleus [70]. Moreover, UVB enhances the expression of KAP1, an inhibitor of the formation of the Nrf2–Keap1–Cul3 complex, required for proteasomal degradation of the transcription factor, which is not entirely cancelled by the microalgae extract. Thus, Nrf2 is less likely to be targeted for degradation by the 26S proteasome, promoting the maintenance of elevated levels of this factor.

The elevated level of HO-1 observed after UVB irradiation and maintained after using the microalgae extract suggests that there is no degradation of this protein, which has not only antioxidant but also anti-inflammatory effects [80]. Remarkably, heme oxygenase-1 (HO-1) is pivotal in the crosstalk between Nrf2 and NF-κB, and plays a crucial role in regulating inflammatory responses [81]. This suggests that the UVB irradiation of keratinocytes not only changes the expression of the transcription factor Nrf2, but also modifies the microenvironment of the pro-inflammatory factor NFκB, increasing the expression of both its subunits (p52 and p65). The use of *Nannochloropsis oceanica* lipid extract only partially prevented this increase. However, keratinocytes treated with microalgae extract alone showed a slight increase in the expression of both NFκB subunits (Figure 8). On the other hand, the use of the extract alone or after UVB irradiation decreased the expression of TNFα (Figure 8), an interleukin that is the primary product of NFκB transcriptional activity. This may be due to the observed upregulation of such NFκB activators as NLRP3, PGAM5, and p62 (Figure 8). The expression of the main NFκB inhibitor, IκB, was decreased by UVB radiation (Figure 8), while it was increased after treatment with the *Nannochloropsis oceanica* lipid extract. Inhibition of the expression of factors associated with the activation of inflammation, such as NLRP3 and PGM5 (Figure 8), with the simultaneous activation of IKK components, especially IKKβ, whose expression was downregulated by UVB radiation, and p62 proteins, indicates a direct link between the NFκB and Nrf2 pathways. Thus, it can be suggested that the increased expression of Nrf2 and HO-1, especially in keratinocytes irradiated after incubation with the microalgae extract, inhibits the activation of the NLRP3 inflammasome, by reducing the production of ROS, which mediates the secretion of pro-inflammatory cytokines, such as IL-1B, responsible for inducing cell death [82]. Also, the extracts seem to prevent the UVB-induced nuclear translocation of NFκB(p52) (Figure 9). This highlights the potential anti-inflammatory effects of microalgae extract in protecting keratinocytes from UVB-induced damage.

Under oxidative stress conditions, such as UVB radiation, the transcription factor NFκB is activated in the canonical pathway in response to increased levels of cytokines, including TNFα [83]. This is accompanied by a decrease in the level of the NFκB inhibitor, IκB, due to reduced levels of the IKKβ and IKKα kinase inhibitors (Figure 8). As a consequence, NFκB is released and translocated to the nucleus (Figure 9), leading to the transcription of pro-inflammatory mediators, including TNFα. The transcriptional efficiency of NFκB is also influenced by the elevated level of 4-HNE (Figure 10), which inhibits the NFκB-IκB complex [84]. After treatment with the microalgae extract, the expression of IKKβ/α increases (Figure 8), and the level of 4-HNE decreases (Figure 10), ultimately promoting an increased level of the IκB inhibitor, which partially inhibits the transcriptional activity of NFκB, as observed with TNFα. Furthermore, the increased transport to the nucleus of NFκB(p52) after UVB irradiation and incubation with the extract (Figure 9) promotes the import of Keap1 into the nucleus, where it terminates Nrf2 gene transcription by exporting this factor back to the cytoplasm [85].

### Future Perspectives and Limitations

The exposure of skin cells to UV radiation, which is a component of solar radiation, leads to the disruption of cellular metabolism, resulting in an imbalance of redox homeostasis and pro-inflammatory reactions that contribute to various pathological conditions. Natural products, including a lipid extract derived from the microalgae *Nannochloropsis oceanica*, have been identified as potential agents to soothe skin inflammation. Therefore, the results obtained in this study contribute to expanding the possibilities of skin protection by understanding the mechanisms of action of the applied protector. However, the most important limitation of this assumption is that the present study allows for a thorough examination of the modified pathways in individual cell lines, whereas the response of epidermal keratinocytes in vivo in complex skin structures can be modified by metabolic interactions with other types of cells present in the skin, including keratinocytes–melanocytes–fibroblasts, as well as immune cells. In addition, the tests carried out provide an answer only to interactions in one plane, and not in a spatial arrangement, as is the case in the skin. Therefore, it can be assumed that the use of 3D culture would give more realistic results. It should also be remembered that the lipid microalgae extract is a mixture of various compounds, which is very beneficial due to the possibility of a broad metabolic response. Therefore, suggestions for practical use in relation to the skin will only apply to an identically prepared extract only from a specific type of algae. 

## 4. Materials and Methods

### 4.1. Microalgal Material

*Nannochloropsis oceanica* biomass was spray-dried and supplied by Allmicroalgae, Natural products S.A. located in Rua 25 de Abril s/n 2445-413 Pataias, Portugal. 

This microalga was cultivated in Guillard’s F2 culture medium adapted to the local water [86]. A magnesium mixture (Necton, Olhão, Portugal) and NaCl (Salexpor, Coimbra, Portugal) at 30 g·L^−1^ salinity were supplemented. *Nannochloropsis oceanica* was cultivated in 5 L flask reactors under continuous 700 μmol photons·m^2^·s^−1^ light exposure from 7 to 15 days. Five 5 L flask reactors were used to inoculate one 0.1 m^3^ L outdoor flat panel (FP) reactor, later scaled up to 1 m^3^ FPs. Four FPs were used as the inoculum of a 10 m^3^ tubular photobioreactor (PBR). The reactor was exposed to ambient light and temperature conditions until the stationary phase was reached. A sprinkler-like irrigation system was used to keep the temperature of the PBR below the maximum limit. The pH was kept constant with pulse injections of CO_2_. Microalgae at approximately 50 g·L^−1^ were dried by atomization in a spray-dryer with an evaporation capacity of 150 kg water·h^−1^. Drying was quickly achieved with an air stream at 215 ± 5 °C. The outlet air temperature with the biomass powder was 92 ± 3 °C. The powder was obtained through a cyclone and stored protected from light and humidity.

### 4.2. Microalgae Lipid Extracts

Lipid extraction of *Nannochloropsis oceanica* was performed using a modified Folch method as described previously [43,87]. Briefly, lipids were extracted using a solvent mixture of dichloromethane:methanol (2:1, *v*/*v*) that was added to 25 mg of biomass. The samples were vortexed and centrifuged at 670× *g* for 10 min. The supernatant was then collected, and this process was repeated three more times. The combined supernatants were dried under a stream of nitrogen. The extracts were then re-dissolved in dichloromethane and methanol and vortexed well, and Mili-Q water was added. Phase separation was attained after centrifugation (670× *g* for 10 min) and the organic phase was collected. The aqueous phase was re-extracted two more times. The lipid extract was obtained by combining the organic phases. The lipid content was determined gravimetrically.

The lipid profile of the obtained *Nannochloropsis oceanica* extracts was characterized by hydrophilic interaction liquid chromatography coupled with high-resolution mass spectrometry (HILIC-MS) and tandem MS (MS/MS) using a Q-Exactive hybrid quadrupole Orbitrap mass spectrometer (Thermo Fisher Scientific, Bremen, Germany) as reported previously [43]. Briefly, two mobile phases, H_2_O/CH_3_CN/CH_3_OH (25:50:25, *v*/*v*/*v*) with 5 mM ammonium acetate (mobile phase A) and CH_3_CN/CH_3_OH (60:40, *v*/*v*) with 5 mM ammonium acetate (mobile phase B), were used in the following gradient: started at 95% B (0–2 min), followed by a linear decrease to 52% B in 8 min, and a further linear decrease to 35% B in 5 min, followed by a 2 min maintenance period and return to initial conditions in 3 min and re-equilibration of the column (10 min). 

Samples (40 μg of lipid extract) were mixed with eluent (95% of B and 5% of A) and lipid standards mixture (dMPC—0.04 μg, LPC—0.04 μg, dMPE—0.04 μg, dMPG—0.024 μg and Cer(17:0/d18:1)—0.04 μg), and 5 μL of this mixture was injected into the Ascentis^®^ Express microbore column (10 cm × 2.1 mm, 2.7 μm; Sigma-Aldrich) at 35 °C with a flow-rate of 200 μL min^−1^. The mass spectrometer operated simultaneously in positive (ESI 3.0 kV) and negative (ESI −2.7 kV) modes, with an automatic gain control (AGC) target of 1 × 10^6^ and high resolution (70,000). The sheath gas flow rate was 20 U, and the capillary temperature was 250 °C. MS/MS determinations were performed with collision energy™ (CE) between 25, 30, and 35 eV and a resolution of 17,500. Data acquisition was carried out using the Xcalibur data system (V3.3, Thermo Fisher Scientific, Waltham, MA, USA). The identification of molecular species of polar lipids was based on LC retention time, mass accuracy (<5 ppm), and detailed structural information inferred by MS/MS data [43].

### 4.3. Cell Culture

Human immortalized keratinocytes CDD 1102 KERTr (CRL2310), obtained from the American Type Culture Collection ATCC^®^ (Manassas, VA, USA), were used in the experiments. Cells from passage 8 were cultured in a humid atmosphere of 5% CO_2_ and a temperature of 37 °C. The growth medium was prepared as follows: keratinocyte–SFM medium supplemented with 1% bovine pituitary extract (BPE) and antibiotics: 50 U/mL penicillin and 50 ug/mL streptomycin. All experiments were performed under sterile conditions, including sterile plastics, and cell culture reagents purchased from Gibco (Grand Island, NY, USA).

The keratinocytes, after reaching 70% confluency, were exposed to UVB radiation. The radiation dose was 60 mJ/cm^2^ (312 nm, power density at 4.08 mW/cm^2^) (Bio-Link Crosslinker BLX 312/365, Vilber Lourmat, Eberhardzell, Germany), which corresponded to approximately 70% of cell survival as measured by the MTT (3-(4,5-dimethylthiazol-2-yl)-2,5-diphenyltetrazolium bromide) method [88] and, as previously shown, leads to the activation of prooxidative conditions [34]. To avoid heat stress, cells were irradiated in cold PBS (phosphate-buffered saline, 4 °C) and the distance of the plates from lamps was constantly maintained at 15 cm. 

After irradiation, the cells were treated with a lipid extract from *Nannochloropsis oceanica* algae in 0.1% DMSO (dimethyl sulfoxide) at concentrations ranging from 1 µg/mL to 1 mg/mL for 24 h without washing under standard conditions. Control cells were incubated in parallel for 24 h with algae extracts under standard conditions (without irradiation) in a medium containing the lipid extracts from *Nannochloropsis oceanica* algae in 0.1% DMSO. The algae extract concentration selected for other experiments (3 µg/mL) did not induce changes in cell viability compared to control cells measured by the MTT test [88]. After 24 h incubation, all cells were washed with PBS, harvested by scraping in cold PBS and, after disintegration, centrifuged, and the resulting solution was used for biochemical assays. The total protein content of the cell lysate was measured using the Bradford assay [89].

### 4.4. Transmembrane Transporters Activities

The activity of membrane ABC cassette transporters (multidrug resistance protein 1 (MDR1), multidrug resistance-associated protein (MRP), breast cancer resistance protein (BCRP)) was determined using a multidrug resistance assay according to the manufacturer’s protocols (eFluxx-ID Multidrug resistance assay kits, Enzo LifeSciences, Farmingdale, NY, USA). Keratinocytes were incubated in dark 96 well plates in PBS buffer for 5 min (37 °C) with inhibitors of ABC transporters resuspended in DMSO (inhibitors of MDR1, MRP and BCRP were verapamil, MK-571 and novobiocin, respectively). The final concentration of DMSO in the buffer was approx. 1%, as well as in control experiments, without inhibitors (containing only PBS and DMSO). Next, EFLUXX-ID^®^ green detection reagent was added, and cells were incubated for 30 min (37 °C). Luminescence was measured (λ_ex_485 nm/λ_em_535 nm) using an EnSpire 2300 Multilable Reader (Perkin Elmer, MA, USA). The activities of MDR1, MRP and BCRP were normalized to the total level of protein, and the final results were expressed as a percentage of the activity of transporters from treated cells (with UVB and/or algae extract) compared to control cells.

### 4.5. ROS Generation

The superoxide anion generation was measured using stable nitroxide CM-radicals formation and detection with an EPR spectrometer (Noxygen GmbH/Bruker Biospin GmbH, Ettlingen, Germany) [90]. Following keratinocytes treatment with UVB and/or algae extract, live cells were incubated with 200 µM CMH (1-hydroxy-3-methoxy-carbonyl-2,2, 5,5-tetrame-thylpyrrolidine) in 36.6 °C for 20 min. During this time the selective interaction of the spin probes formed with ROS a stable nitroxide CM-radical with a half-life of 4 h. Next, the samples were transferred to ice to stop further reactions. Thus, ROS formation was measured from the kinetics of the nitroxide accumulation according to the electron spin resonance amplitude of the low field component of ESR spectra. The results are shown as micromoles of superoxide anions generated per minute and normalized per mg of protein.

### 4.6. Pro-Oxidant Enzymes Activities

NADPH oxidase (NOX-EC 1.6.3.1) activity was examined by the luminescent method using lucigenin as a luminophore [91]. Cell lysates were added to phosphate buffer (50 mM, room temperature, pH 7.0) supplemented with ethylene glycol-bis(2-aminoethylether)-N,N,N′,N′-tetraacetic acid (1 mM, EGTA), saccharose (150 mM), NADPH (10 mM) and lucigenin (1 mM). The luminescence was measured for one minute immediately after mixing the sample (Microplate reader Infinite M200, TECAN Trading AG, Männedorf, Switzerland). The amount of enzyme required to release 1 nmol of superoxide anion radicals per minute was equivalent to one unit of NOX activity and is expressed in relative luminescence units (RLU). Enzyme-specific activity is shown in relative luminescence units (RLU) per mg of protein.

Xanthine oxidase (XO-EC1.17.3.2) activity was estimated by uric acid formation from xanthine [92]. Cell lysates were added to phosphate buffer (50 mM, room temperature, pH 7.5) supplemented with xanthine (150 µM). Next, the changes in absorbance at 290 nm were measured for one minute immediately after mixing the sample (Microplate reader Infinite M200, TECAN Trading AG, Männedorf, Switzerland). One XO unit was defined as the amount of enzyme required to release 1 µM uric acid per minute. Enzyme activity is shown in microunits per mg of protein.

### 4.7. Antioxidant Enzymes Activity and Low-Molecular Antioxidants Levels

The activity of superoxide dismutase (SOD1 (Cu,Zn-SOD-EC.1.15.1.1)) [93] and manganese-dependent superoxide dismutase (SOD2 (Mn-SOD–EC.1.15.1.1)) was determined by the spectrophotometric method (with wavelength for absorbance reading at 480 nm) [94]. The measurement was performed in comparison to the control in which a carbonate buffer solution containing 100 µM EDTA solution at pH = 10.2 was used. The specific activity of superoxide dismutase was calculated and is presented in units per mg of protein.

Catalase (CAT—EC 1.11.1.9) activity was determined spectrophotometrically (at 240 nm) by the estimation of hydrogen peroxide decomposition rate [95]. The amount of the enzyme catalyzing 1 µmol of hydrogen peroxide decomposition to water and oxygen within 1 min was described as one unit of CAT activity. Enzyme-specific activity is expressed in units per mg of protein.

The activity of glutathione peroxidase (GSHPx–EC.1.11.1.6) was assessed based on GSSG reduction with the oxidation of NADPH to NADP [96]. The oxidation of 1 µmol NADPH min^−1^ at 25 °C and pH 7.4 was defined as one GSHPx activity unit, which allowed us to observe the reduction in absorption at 340 nm. The specific activity of glutathione peroxidase was calculated and is presented in milliunits per mg of protein.

To determine the activity of glutathione reductase (GSSG-R-EC.1.6.4.2), an indirect method was used, involving the reduction of GSSG due to the oxidation of NADPH to NADP, which reduces absorption at 340 nm [97]. The decrease in absorbance was assessed within 1 min against a control containing phosphate buffer (0.02 M; pH 7). The enzyme-specific activity was calculated and is presented in milliunits per milligram of protein.

The reduced glutathione (GSH) level was measured using capillary electrophoresis (CE) [98] with separation compounds on a 47 cm capillary operated at 27 kV. UV detection was set to 200 nm. The calibration curve range of 1–120 nmol/mL (r^2^ = 0.9984) was used to determine the GSH level, which is expressed in nanomoles per mg of protein.

Thioredoxin reductase activity (TrxR-EC.1.8.1.9) was measured using a commercially available kit (Sigma-Aldrich, St. Louis, MO, USA) based on the NADPH reduction of 5,50-dithiobis(2-nitrobenzoic acid) (DTNB) to 5-thio-2-nitrobenzoic acid (TNB) [99]. One unit of TrxR was defined as the amount of enzyme that caused an absorbance increase (412 nm) per minute per mL at 25 °C and in pH 7. Enzyme-specific activity is expressed in units normalized per milligram of protein.

Thioredoxin (Trx) levels were quantified using the ELISA (enzyme-linked immunosorbent assay) method [99]. The ELISA plates coated with samples were incubated at 4 °C overnight with anti-thioredoxin primary antibody (diluted in 1% bovine serum albumin in phosphate-buffered saline (PBS)) (Abcam, Cambridge, MA, USA). After washing, the plates were incubated at room temperature for 30 min with a peroxidase-blocking solution (3%H_2_O_2_, 3% fat-free dry milk in PBS). Then, goat anti-rabbit secondary antibody (Dako, Carpinteria, CA, USA) was added for 1 h. Next, a chromogen substrate solution (0.1 mg ml^−1^ TMB, 0.012% H_2_O_2_) was added to each well. The reaction was stopped by sulfuric acid. Absorbance was read at 450 nm with the reference filter set to 620 nm. The level of thioredoxin is expressed in μg per mg of protein.

### 4.8. Proteins Levels

Protein expression measurement was performed using ELISA [100]. Lysates of keratinocytes were applied to ELISA plate wells (Nunc Immuno Maxi Sorp, Thermo Scientific, Waltham, MA, USA). Plates with attached proteins were incubated at 4 °C for 3 h with a blocking solution (5% fat-free dry milk in carbonate binding buffer). After washing with PBS with 0.1% Tween 20, samples were incubated at 4 °C overnight with appropriate primary antibodies against NFκB (p65), NFκB (p52), TNFα (Sigma-Aldrich, St. Louis, MO, USA), HO-1 (Invitrogen, Walthman, MA, USA), p21 (Abcam, Cambridge, MA, USA), (host: mouse), Nrf2, Bach1, PGAM5, IKKβ (Sigma-Aldrich, St. Louis, MO, USA), phospho-Nrf2 (Ser40) (Invitrogen, Waltham, MA, USA), p62, KAP1 (Sigma, Shanghai, China), MAPK (Cell Signalling Technology, Danvers, MA, USA), IKKα (Thermo Fisher, Waltham, MA, USA) (host: rabbit), Keap1 (Sigma-Aldrich, St. Louis, MO, USA), and NLRP3 (Sigma Aldrich Santa Cruz, TX, USA) (host: goat). All antibodies were used at a dilution of 1:1000. Next, following washing (PBS with 0.1% Tween 20), the plates were incubated for 30 min with peroxidase blocking solution (3% H_2_O_2_, 3% fat-free dry milk in PBS) at room temperature. Goat anti-rabbit/mouse EnVision+ Dual Link/HRP solution (1:100) (Agilent Technologies, Santa Clara, CA, USA) was used as a secondary antibody. After 1 h of incubation at room temperature, the secondary antibody was removed and the plates were incubated with chromogen substrate solution (0.1 mg/mL TMB, 0.012% H_2_O_2_) for 40 min. The reaction was stopped by adding 2 M sulfuric acid and absorption was read within 10 min at 450 nm and automatically recalculated from standard curves prepared for each protein (NFκB (p65, 1–5 µg/mg prot), PGAM5 (1–5 µg/mg prot), Bach1 (1–5 µg/mg prot) (OriGene, Rockville, MD, USA), NLRP3 (1–5 µg/mg prot), p-21 (1–5 µg/mg prot), IKKα (1–5 µg/mg prot), IKKβ (1–5 µg/mg prot), KAP1 (1–5 µg/mg prot) (Abcam, Cambrige, UK), TNFα (1–5 µg/mg prot)(Merck, Darmstadt, Germany), NFκB (p52, 1–5 µg/mg prot) (LSBio, Seattle, WA, USA), HO-1 (1–5 µg/mg prot) (Enzo Life Sciences, Farmingdale, NY, USA), p38 (1–5 µg/mg prot) (Bio-techne, Minneapolis, MN, USA), Keap1 (1–5 µg/mg prot) (Sino Biological Europe, Düsseldorfer, Eschborn, Germany), Nrf-2 (1–5 µg/mg prot), phospho-Nrf2 (Ser40) (1–5 µg/mg prot) (Belgium Gentaur BV, Kampenhout, Belgium), p62 (1–5 µg/mg prot) (Boster Biological Technology, Pleasanton, CA, USA), MAPK (1–5 µg/mg prot) (Cell Signaling Technology, Danvers, MA, USA), DPP3 (MilliporeSigma, Shanghai, China)).

### 4.9. Protein Localization

Cells were seeded in black, 96-well, clear-bottom tissue culture plates, and after UVB irradiation and 24 h incubation, were rinsed with PBS and fixed with methanol. Following permeabilization with 0.1% Triton X-100 at room temperature, non-specific binding places were blocked by incubation in 3% FBS. Next, cells were incubated with either anti-Nrf2 rabbit polyclonal antibodies or anti-NFκB (p52) mouse polyclonal antibodies (Sigma-Aldrich, St. Louis, MO, USA) for 1 h at room temperature. Goat anti-rabbit/mouse EnVision+ Dual Link/HRP solution (1:100) (Agilent Technologies, Santa Clara, CA, USA) was used as a secondary antibody. Nuclei were stained with Hoechst (2 µg/mL) and analyzed using a Nikon Eclipse Ti fluorescence microscope with a DS-Qi2 camera (Nikon Instruments Inc., Tokyo, Japan) under 488 nm excitation and 525 nm emission wavelength.

### 4.10. Quantification of Lipid Peroxidation Products

4-hydroxynonenal (4-HNE) was measured by GC/MS in selected ion monitoring (SIM) mode, with the O-PFB-oxime or O-PFB-oxime-TMS derivatives using benzaldehyde-D6 as an internal standard [101]. Aldehyde was analyzed using a 7890A GC–7000 quadrupole MS/MS (Agilent Technologies, Palo Alto, CA, USA) equipped with an HP-5ms capillary column (30 m length, 0.25 mm internal diameter). Target ions with an *m*/*z* 333.0 and 181.0 for 4-HNE-PFB-TMS and *m*/*z* 307.0 for IS derivatives were selected. The obtained results were normalized and are presented for milligrams of protein.

The 8-Isoprostaglandin F2 (8-isoPGF2) level was measured using LC-MS analysis performed on an Agilent 1290 LC coupled with an Agilent 6460 triple quadrupole mass spectrometer (Agilent Technologies, Palo Alto, CA, USA) in negative multiple reaction mode [102] and the transition *m*/*z* 353→193 was selected. SEP-PAK C18 columns were used for sample purification.

### 4.11. Protein-4-HNE Adducts Determination

Protein oxidative modifications in keratinocytes were estimated as 4-HNE-protein adducts level measured by ELISA method described above [100] using anti-4-HNE-protein monoclonal mouse antibody (Invitrogen, Burlington, ON, Canada) and goat anti-rabbit/mouse EnVision+ Dual Link/HRP solution (1:100) (Agilent Technologies, Santa Clara, CA, USA) as a secondary antibody. The concentrations of 4-HNE–protein adducts were determined using a calibration curve range of 0.5–25 pmol/mg of BSE (r^2^ −0.9987) and converted to 1 mg of protein.

### 4.12. Statistical Analysis

Data were assessed for normal distribution using the Shapiro–Wilk test and are presented as mean ± standard deviation (SD) for n = 5. Student’s *t*-test was employed for data analysis. All experiments were conducted on cell lines, and technical repetitions were used as replicates, which do not reflect biodiversity. A *p*-value of <0.05 was considered statistically significant.

## 5. Conclusions

In conclusion, it can be postulated that the lipid components extracted from *Nannochloropsis oceanica* do not induce significant changes in the keratinocyte metabolism under non-stressed conditions, but effectively hinder the pro-oxidative and pro-inflammatory effects of UVB radiation in these cells. The findings presented in this study suggest that this effect is achieved through a reduction in reactive oxygen species (ROS) generation, as well as the activation of enzymatic antioxidant systems, including glutathione (GSH) and thioredoxin (Trx)-dependent enzymes. Additionally, the protective action of the lipid extract of *Nannochloropsis oceanica* is also linked to the activation of the Nrf2 pathway, as well as the suppression of the pro-inflammatory activity of the transcription factor NFκB. Furthermore, its antioxidative action inhibits UVB-induced lipid peroxidation, thereby silencing the pro-inflammatory signaling that relies on the products of oxidative lipid metabolism, such as 8-isoPGF2α or 4-HNE-protein adducts. As a result, the natural metabolism of skin cells is stabilized even when exposed to harmful UVB radiation.

## Data Availability

All data generated or analyzed during this study are included in this published article.
